# Empowering fungal infection research with single-cell RNA sequencing

**DOI:** 10.1038/s42003-026-10304-x

**Published:** 2026-05-29

**Authors:** Renata Toth, Florabelle Ruano Cabarrubias, Zsolt Czimmerer, Kitti Vecsernyes-Nagy, Attila Gacser

**Affiliations:** 1 Department of Biotechnology and Microbiology, University of Szeged, Szeged, Hungary; 2 Institute of Genetics, HUN-REN Biological Research Centre, Szeged, Hungary; 3 HCEMM-SZTE Pathogen Fungi Research Group, University of Szeged, Szeged, Hungary; 4 HUN-REN-SZTE Pathomechanisms of Fungal Infections Research Group, University of Szeged, Szeged, Hungary; 5 Competence Centre for Molecular Biology, Bionics and Biotechnology, University of Szeged, IKIKK, Szeged, Hungary

**Keywords:** Fungal infection, High-throughput screening, Fungal host response

## Abstract

Fungal pathogens represent a rising global concern with increasing impacts on human health and food security. Despite their significance, research on fungal infections continues to lag behind other infectious diseases, hindering diagnostic and treatment advances. Single-cell RNA sequencing (scRNA-seq) is a powerful tool widely used to identify biomarkers and targets of intervention in various fields, including host-pathogen research. Owing to its ability to resolve cellular heterogeneity, scRNA-seq has been successfully applied in host-viral and host-bacterial studies, providing in-depth insights into the mechanisms of pathogenesis. Recently, this method has also been increasingly adopted in fungal infection research. Here, we provide a brief overview, that summarizes key findings and offers in-depth insights into the dynamics of host–fungal pathogen interactions uncovered through this approach. The review also addresses current limitations, gaps and future directions, encouraging researchers for the broader adoption of single-cell technologies in this field.

## Abstract (web summary)

It is summarized how scRNA-seq enhances our understanding of host–fungal pathogen interactions by highlighting recent mechanistic insights, current knowledge gaps, and the future of single-cell technologies in the field.

## Introduction

Communicable diseases pose a major global burden. While viral and bacterial infections are extensively studied and addressed, fungal infections receive comparatively less attention and remain an underinvestigated field^1^. To address this issue, the recently published WHO Fungal Pathogens Priority List^2^ underscored the growing severity of human fungal infections. Concurrently, a recent commentary highlighted the expanding spread of crop fungal infections^3^, posing a risk to global food security, indirectly affecting public health and quality of life. The rise of antifungal resistance and limited treatment options further signifies the need for a deeper understanding of fungal pathogenesis and host responses.

In response to these calls, it is anticipated that fungal pathogens will gradually gain better overall recognition, although the field currently lags behind compared to other infectious diseases. This is reflected not only in the smaller body of available literature and funding dedicated to the field^4^, but also in the delay in adapting novel methods or platforms from viral and bacterial research to fungal investigations^5,6^. An example is the relatively slow adoption of emerging high-throughput technologies, such as single-cell RNA sequencing (scRNA-seq), to pathogenic fungal research^6^.

Since their introduction in fundamental research, Next Generation Sequencing (NGS) technologies have advanced infectious disease research by enabling comprehensive genomic and transcriptomic profiling. Transcriptomics is particularly valuable in infection biology, as it provides a powerful tool for capturing dynamic changes in gene expression during infection, offering insights into host immune responses and pathogen invasion strategies. However, conventional bulk RNA sequencing collects and combines signals from multiple, often highly diverse cell populations, which might mask the complexity of cellular responses and hide rare subpopulations with a potentially unique role^6–8^.

To overcome these limitations, scRNA-Seq has emerged as a novel method that measures gene expression at the level of individual cells^9,10^. This method has been successfully applied in various forms, in diverse fields from cancer biology to immunology^11–17^. In the context of host-pathogen research, scRNA-seq enabled unique insights into pathogen heterogeneity, rare immune cell subsets, permissive and resistant host cell subsets, or individual gene expression patterns^18–21^. This has led to significant discoveries in viral and bacterial infections, deepening our understanding of immune evasion, tissue tropism, and pathogen adaptation, as summarized in previous studies^22–27^.

**Fig. 1:**
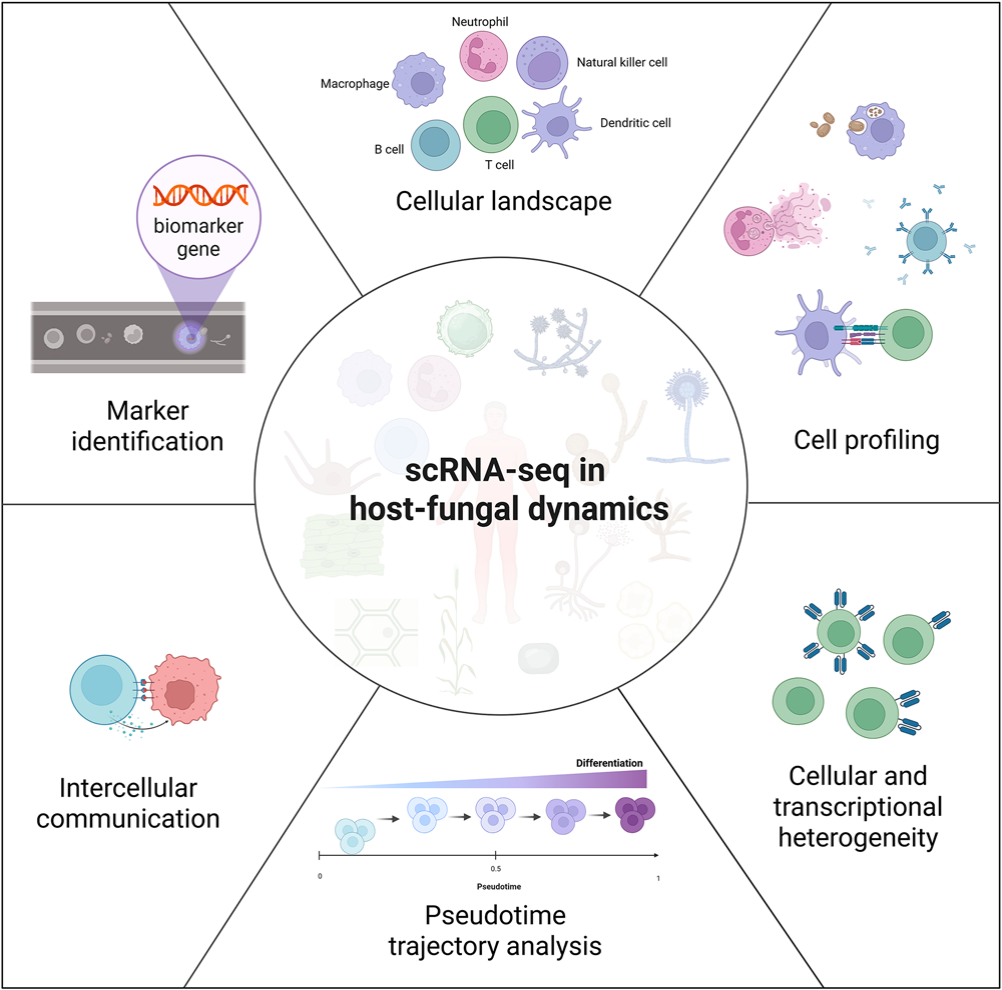
Overview of the potential of scRNA-seq to dissect the host–fungal interplay. Summary of the six primary discovery areas: cellular landscape, profiling, marker genes, cell-to-cell communication, developmental trajectories, and transcriptomic heterogeneity. Figure 1 was created in BioRender. Gacser, A. (2026) https://BioRender.com/pg212x0.

More recently, the use of scRNA-seq in fungal infection studies—both human and plant fungal pathogen infections—is gaining rapid prominence (Table 1). This approach offers the potential to better understand the complex interactions between fungal pathogens and host cells at the single-cell level. Using scRNA-seq, these studies identified host cell populations responding to fungal infections (cellular landscape) and characterized their behavior (cellular profiling). They also captured responses that varied within the identified populations (cellular or transcriptional heterogeneity) and predicted how they might evolve during disease progression (pseudotime trajectory analysis). The studies also mapped potential interactions between these populations (intercellular communication) and uncovered diagnostic signatures (marker identification) (Fig. 1).

**Table 1:** Host–fungal pathogen interaction studies using scRNA-sequencing

Disease	Host	Fungal pathogen	Purpose of scRNA-Seq in the study	scRNA-Seq. Method	Additional Seq. method	Year	Ref.
Aspergillosis	*Homo sapiens*	*Aspergillus fumigatus*	Identification of *A. fumigatus-*specific airway epithelial responses in a pseudostratified epithelial layer model (hAEC)	10× Genomics	-	2025	^33^
	*Homo sapiens*	*Aspergillus* spp.	Characterization of innate (monocyte/macrophage/PMN) and adaptive (T cells) immune responses during COVID-19-associated pulmonary aspergillosis	10× Genomics	-	2024	^36^
	*Mus musculus*	*Aspergillus fumigatus*	Examining the role of DCs in allergic airway inflammation due to *A. fumigatus* exposure	10× Genomics	-	2025	^37^
Coccidiomycosis	*Homo sapiens*	*Coccidioides posadasii*	Identification of *C. posadasii-*specific airway epithelial responses in a pseudostratified epithelial layer model (hAEC)	10× Genomics	-	2025	^33^
	*Mus musculus*	*Coccidioides posadasii*	Exploring dynamic changes in the lung immune landscape in an intranasal mouse model of *C. posadasii* infection	10× Genomics	Spatial transcriptomics	2024	^41^
Pneumocystis pneumonia	*Mus musculus*	*Pneumocystis murina*	Analysis of T cell dynamics over the course of *Pneumocystis murina* infection in the lungs of mice	10× Genomics	scTCR-seq	2021	^69^
	*Rattus norvegicus*	*Pneumocystis carinii*	Characterization of the life cycle of a host-obligate fungal pathogen, (*P. carinii*) recovered from rat bronchoalveolar lavage fluid	10× Genomics	-	2025	^71^
Cryptococcosis	*Mus musculus*	*Cryptococcus neoformans*	Identification of distinct *C. neoformans*-responsive PMN populations in mouse lungs	10× Genomics	-	2021	^45^
	*Mus musculus*	*Cryptococcus neoformans*	Revealing alveolar macrophage (AM) functional heterogeneity in *C.* *neoformans-*infected murine lungs	10× Genomics	Bulk RNA-seq, ATAC-seq	2021	^46^
	*Homo sapiens*	*Cryptococcus neoformans*	Identification of host protective APC subpopulations in human bronchoalveolar lavage fluids in response to *C. neoformans* presence	10× Genomics	-	2022	^48^
Candidiasis	*Homo sapiens*	*Candida albicans*	Identification of a candidaemia susceptibility biomarker	10× Genomics	Bulk RNA-seq, GWAS	2020	^56^
	*Homo sapiens*	*Candida albicans*	Meta-analysis of blood transcriptome studies to reveal network interplay between TLR and IFN signaling during candidiasis	scRNA-seq dataset from (56)	Bulk RNA-seq, microarray	2021	^57^
	*Mus musculus*	*Candida albicans*	Exploring host-pathogen co-transcriptional dynamics during mouse BMDM – *C. albicans* interactions	SMART-Seq2	Bulk RNA-seq	2019	^58^
	*Mus musculus*	*Candida albicans*	Assessing the effect of systemic candidiasis on host responses within the adrenal gland in a mouse model	10× Genomics	-	2022	^60^
	*Mus musculus*	*Candida auris*	Revealing skin immune and non-immune host responses against *C. auris* and potential immune evasion strategies	10× Genomics	-	2024	^61^
	*Homo sapiens*	Clinical sample: *C. albicans* isolated	Characterization of immune and non-immune microenvironmental changes in tongues of chronic hyperplastic candidiasis patients	Singleron Matrix	-	2024	^52^
General plant fungal disease and Anthracnose	*Arabidopsis thaliana*	*Colletotrichum higginsianum*	Assessing the role of NLR receptors and GSL metabolism during *C. higginsianum* infection of leaves	10× Genomics	Bulk RNA-seq	2023	^74^
Stalk rot	*Zea mays*	*Fusarium verticillioides*	Identification of immune regulatory networks in *F. verticillioides*-infected maize roots	10× Genomics	Bulk RNA-seq	2023	^79^
Head blight	*Triticum aestivum*	*Fusarium graminearum*	Characterization of *F. graminearum*-specific host responses and susceptibility causes in wheat coleoptiles	BD Rhapsody	LCM-based bulk RNA-seq	2025	^84^
Rice blast	*Oryza sativa*	*Magnaporthe oryzae*	Identification of cell-specific and multidimensional host responses in *M. oryzae-*infected rice leaves	DNBelab C Series	Spatial transcriptomics	2025	^87^

Considering these six aspects of scRNA-seq findings—where applicable—in this review, we provide an overview of how scRNA-seq has helped advance our understanding of host-fungal pathogen interactions. We highlight significant findings and conclusions that emerged, thereby illustrating how this technology has advanced our understanding of the complex dynamics between different hosts and invading fungi.

## Human-fungal pathogen interactions

Human fungal pathogens are a growing global health concern, with an estimated 1 billion affected individuals, approximately 150 million life-threatening infections and 1.7 million deaths annually^28^. The growing number of immunosuppressed patients, climate change and the rise of drug-resistant strains all contribute to their emergence. The responsible species commonly affect immunocompromised individuals, such as those with an underlying disease, surgical patients, or patients receiving prolonged immunosuppressive/antimicrobial treatment, although immunocompetent hosts can also be infected^29^. Such infections manifest in either systemic or superficial forms. Given that invasive fungal infections often present non-specific clinical symptoms that mimic bacterial infections, understanding their development and identifying superior markers for early diagnosis and therapy are critical. Below, we summarize recent scRNA-seq findings of several fungal pathogenic genera studied to date: *Candida*, *Cryptococcus*, *Aspergillus*, *Pneumocystis*, and *Coccidioides*. Due to the excessive volume of data provided in these studies, we provide only a brief overview of their major findings.

### Aspergillosis

*Aspergillus* species are responsible for pulmonary and invasive infections, known as aspergillosis, primarily in immunocompromised individuals. The most frequently reported species accounting for 60 to 90% of infections is *A. fumigatus*, although *A. flavus, A. niger*, and *A. terreus* are also common clinical isolates^30^. The common route of infection is through the inhalation of airborne spores, leading to either aspergilloma (fungal balls in the lungs or sinuses), allergic bronchopulmonary aspergillosis (approx. >4.8 million affected individuals), chronic pulmonary aspergillosis (approx >1.8 million), or invasive aspergillosis (approx. >2.1 million)^31,32^. scRNA-sequencing was used in Aspergillosis-related studies that investigated human airway epithelial responses to the fungal infection, *A. fumigatus (Af)*-induced allergic airway inflammation, and COVID-19-associated pulmonary aspergillosis (CAPA).

#### Airway epithelial responses to *Af*-infection

##### ***Af*-reactive epithelial cell types and their transcriptional responses during pulmonary infection**

To model host responses during pulmonary aspergillosis using an ex vivo approach, Harding et al. generated human lung epithelia (hAECs) from primary basal cells to mimic the cellular environment of the human airway^33^. Following *Af* infection, scRNA-seq identified ciliated cells as the most affected cell type, exhibiting the most prominent transcriptional impact. Hillock, basal, and secretory cells were also heavily impacted, highlighting their potentially important role in anti-*Af* responses.

Based on their transcriptomic profiles, ciliated cells were the most reactive, activating unfolded protein response (UPR) pathways via heat shock protein upregulation (e.g., *HSPA1A/HSPA1B/HSPA8, HSPB1* via *HSF1*; *DDIT4*) potentially to counter pathogen-induced stress. Notably, these cells and nearly all other epithelial cell types strongly induced chemoattractants (mainly to recruit neutrophils for subsequent clearance), establishing the lung epithelium as an early coordinator of innate immune recruitment. Other epithelial populations also showed cell-type-specific responses: hillock cells upregulated genes involved in negative regulation of transcription, basal cells in metabolic and sterol biosynthesis pathways, and secretory cells in UPR activation and sterol biosynthesis. Therefore, scRNA-seq strengthened the link between the lung epithelia and the primary effector cells, neutrophils, in the fight against pulmonary aspergillosis^34,35^.

#### COVID-19-associated pulmonary aspergillosis

##### **Innate immune cell landscapes and heterogeneity in CAPA**

In a COVID-19-associated pulmonary aspergillosis study, comparing CAPA and COVID-19 patients, key shifts were identified in the bronchoalveolar lavage fluid (BALF) immune profile of CAPA patients, with a rise in monocyte/macrophage and epithelial cells, and a parallel decline in neutrophil levels^36^. From the seven neutrophil phenotypic subtypes, a subgroup of immature neutrophils, three subgroups of mature neutrophils, an aged neutrophil, a pre-hybrid neutrophil and a hybrid neutrophil subgroup were identified. Among these, hybrid neutrophils were most abundant in CAPA patients compared to COVID-19 patients.

Hybrid neutrophils in CAPA patients were further characterized, and downregulated pro-inflammatory responses and upregulated iron and zinc sequestration were revealed as key changes, albeit non-canonical functions were also revealed, including enhanced degranulation and MHC class II antigen presentation. Additionally, monocytes and macrophages also displayed a dysfunctional phenotype characterized by the reduced expression of pro-inflammatory mediators and metabolic reprogramming. Upregulated oxidative phosphorylation, downregulated glycolysis and mTOR signaling scores indicate compromised conidial clearance, contrasting with the conventional mTOR-driven glycolytic activation needed for efficient macrophage function^36^. These findings indicate disturbances in neutrophil, monocyte and macrophage functions that could predispose COVID-19 patients to develop CAPA.

##### **Developmental trajectories revealed arrested neutrophil and T-cell maturation**

Pseudotime trajectory analysis (computational ordering of cells along a developmental path) in the CAPA study revealed that the observed reduction in neutrophils in CAPA compared to COVID-19 patients is potentially due to the loss of mature neutrophils, making pre-hybrid and hybrid neutrophils the dominant neutrophil population upon *Af* superinfection^36^. While T cell abundance was similar between CAPA and COVID-19 samples, pseudotime trajectory analysis also suggested arrested T cell differentiation in CAPA. This was marked by reduced CD4+ effector-memory cells with impaired Th1/Th17 potential and a depleted exhausted CD8 + T cell population. Consequently, this arrested maturation highlights another potential immune failure that may lead to higher mortality in CAPA compared to COVID-19.

#### *Af*-induced allergic pulmonary inflammation

##### **DC cellular and transcriptional heterogeneity is linked to airway allergy**

In a murine model of fungi-induced allergic airway inflammation, using scRNA-seq, Cook et al. studied the role of dendritic cell (DC) subsets in disease pathology^37^. Comparisons between naïve mice and those exposed to *Af* spores revealed seven transcriptionally distinct DC populations: three conventional DC populations (cDC1, cDC2, and a CCR7+ “Mreg-like” DC subset) and four monocyte/inflammatory DC (Mo, Inf-DC1, Inf-DC2, Inf-DC3) subsets with distinct monocyte-derived activation signatures. Notably, the Mo, Inf-DC1, Inf-DC2, and Inf-DC3 subsets expanded specifically after *Af* exposure.

Although mainly the Inf-DC subsets expanded, most DC populations—including cDC1, cDC2, CCR7 + , Inf-DC2, Inf-DC3, and Mo—showed late-stage upregulation of genes involved in oxidative phosphorylation, antigen processing, and phagocytosis. Notably, these DC populations exhibited the highest enrichment for gene programs associated with Th2 and Th17 responses, identifying them as candidate drivers of fungal allergic airway inflammation. Further experimental analysis revealed Mgl2 + cDC2s, coordinating allergic airway type 2 inflammation.

### Coccidioidomycosis

*Coccidioidomycosis*, or Valley fever, is a fungal infection primarily caused by *C. immitis* or *C. posadasii* endemic to the southern parts of North America, Central-and South-America^38^. However, its geographic range has been reported to expand, with an estimated 350,000 cases annually in the US alone^39^. Following the inhalation of airborne arthroconidia from arid soils, approx. 30% of the individuals develop pulmonary coccidioidomycosis, and up to 2% develop disseminated infection. While immunocompromised individuals face the highest risk for dissemination, certain immunocompetent groups also show higher incidence: non-Caucasian ethnicities have intrinsic susceptibility, whereas travelers and outdoor workers face increased environmental exposure^38,40^.

#### Resident cells and immune landscapes in the lungs

Using the same ex vivo hAEC model employed for *Af, C. posadasii* (*Cp*) was also shown to target specific cell types in the lung tissue. Secretory cells exhibited the most robust transcriptional response, with over 6000 differentially expressed genes (DEGs), although hillock, basal, and ciliated cells were also affected^33^.

Another study using a murine model of pulmonary coccidioidomycosis profiled diverse lung cell populations after intranasal arthroconidia inoculation. The study reported that resident cells in the lungs progressively declined over time as immune infiltrates expanded, with a shift in the latter’s composition^41^. Specifically, following a temporary increase, lymphocyte populations (CD4 + , CD8 + , Treg, NK, and B cells) declined, while myeloid cells (neutrophils and monocytes/macrophages) increased as the disease progressed. Subclustering of monocytes/macrophages identified 11 distinct myeloid subtypes, with SPP1+ (osteopontin) macrophages dominating at the late infection stage^41^.

#### Oxidative stress responses and fibrosis dominate in infected-lung tissues

Profiling of the hAECs revealed a pervasive hypoxic stress signature across multiple cell types during infection. As such, secretory/club cells showed upregulation of hypoxia-responsive genes and iron homeostasis genes. Disruption of iron homeostasis further induces hypoxic stress. Inflammatory responses were also upregulated through chemokines and alarmins, which are also heavily induced by hypoxia pathways, indicating a link between hypoxic and immune responses^33^. Such an immune evasion strategy disrupts epithelial barrier function while coordinating inflammatory responses favorable to fungal persistence.

In the murine pulmonary coccidioidomycosis model, infected cells showed lung dysfunction signatures^41^. Several late-expanding neutrophil subgroups displayed distinct profiles: PD-L1+ (inflammatory/apoptotic), circulating (migratory), early B (E2F targets), and early A (degranulation, ROS, NETs). The likewise late-emerging SPP1+ macrophage population was enriched in lung fibrotic, hypoxic, complement, proinflammatory and profibrotic mediator pathways and expressed neutrophil-recruiting chemokines. Notably, fibroblasts, mesenchymal, and endothelial cells also showed enrichment in fibrotic signatures, specifically epithelial-mesenchymal transition (EMT), collagen formation and extracellular matrix organization. Early fibrotic signatures were also mirrored in certain epithelial cells.

These findings suggest that extensive infiltration of diverse neutrophils and emerging SPP1+ macrophages drive late-stage pulmonary coccidioidomycosis, while mesenchymal, fibroblast and epithelial cells, besides SPP1+ macrophages, mediate fibrosis.

#### Monocyte/macrophage and fibroblast communication linked to fibrosis

Assessing cell communication in the in vivo pulmonary coccidioidomycosis model, ligand-receptor expression profiles identified fibroblasts as the dominant cell type associated with angiopoietin-like protein (ANGPTL) and IL-6 signaling pathways, with both linked to lung fibrosis and lung leakage. Notably, monocytes/macrophages were the primary receivers of the ANGPTL and IL-6 fibroblast signals, suggesting a critical intercellular link between fibroblasts and monocytes/macrophages during *Cp* infection, driving disease pathology^41^.

### Cryptococcosis

Cryptococcosis, due to *C. neoformans* and *C. gatti*, is a fungal infection, affecting an estimated 1 million individuals, with 600 000 lethal cases annually worldwide^42^. The disease primarily affects the lower respiratory tract and the central nervous system, with the latter linked to high mortality rates^43^. Following the inhalation of basidiospores from environmental sources, e.g., soil, bird droppings, tree trunk bases, the fungus resides in the lungs and disseminates to other organs, commonly the brain^44^. Although both immunosuppressed and immunocompetent individuals are at risk, immunosuppressed patients, especially those with HIV or receiving immunomodulatory therapy, are prone to cryptococcal meningitis.

#### Neutrophil and alveolar macrophage heterogeneity and profiling

Currently, there is limited information on dynamic cellular landscapes during cryptococcosis. Available studies focus on designated cell types, e.g., neutrophils or professional antigen-presenting cells (APCs) partaking in cryptococcus clearance.

When examining mouse lungs during acute pulmonary cryptococcosis by *C. neoformans* (*Cn*), one study reported the significant expansion of neutrophils^45^. Subsequent analysis revealed three major neutrophil subclusters (I–III), of which cluster II emerged specifically after the infection. Further characterization of this cluster revealed two functionally distinct subsets: IIa (oxidative signature neutrophils, or Ox-PMNs) enriched in iron processing, ROS/RNS, and glycolysis; and IIb (cytokine signature neutrophils, or Cyt-PMNs) enriched in interleukin and cytokine signaling pathways, suggesting different PMN activation states during acute infection.

Using the same dataset, the role of alveolar macrophages (AM) was also assessed in pulmonary cryptococcosis^46^. Comparing uninfected and *Cn*-infected mice lungs, two macrophage branches were identified: homeostatic AMs, predominant in uninfected lungs, and responsive AMs, dominating in infected lungs. Compared to homeostatic AMs, immune receptors and cytokines were upregulated, while lipid metabolism genes were downregulated in responsive AMs, indicating inhibition of normal surfactant metabolism^46,47^. Hence, the study showed that *Cn*-infected lungs contain mostly activated AMs with unique immune receptor profiles.

In another study, scRNA-seq was performed on APCs, derived from human BAL samples, to resolve *Cn*-induced transcriptional heterogeneity^48^. As a result, naive and *Cn*-specific clusters were both identified. However, due to discrepancies in clustering patterns, the exact cell types could not be confidently determined with known reference markers.

#### Pseudotime trajectory analysis revealed protective and metabolic functions of APCs

Lacking clear canonical markers, pseudotime trajectory analysis was used to map functional immune transitions in *Cn*-infected human BAL samples^48^. Two trajectories emerged: a metabolic trajectory driven by mTOR-related mitochondrial genes and a protective trajectory focused on pathogen elimination, based on upregulation of lipid metabolism, immune recognition and antigen processing. The latter progressed through two functional stages: top clusters focused on ‘Recognition and Digestion’ (expressing scavenger receptors, stress sensors, lysosomal indicators, and MHC-II molecules for antigen presentation) and bottom clusters transitioned to 'Cytokine Signaling', or systemic immune coordination (expressing pro-inflammatory cytokines and transcription factors), orchestrating broader protective responses.

#### Intercellular communication between neutrophils and DCs/AMs

To reveal intercellular communication in acute pulmonary cryptococcosis, ligand-receptor interactions were assessed between neutrophils (Ox-PMNs/Cyt-PMNs) and APCs (DCs/AMs)^45^. While both PMN types were predicted to communicate with DCs and AMs, Ox-PMNs and Cyt-PMNs also exhibited unique signature interactions. While Ox-PMNs interacted mainly with DCs (*via Alcam–CD6*), Cyt-PMNs strongly communicated with both DCs and AMs (e.g., via *TNFa-TNFaR2, IL1-a – IL1aR1/R2*). Thus, both PMN subsets contribute to effective *Cn* clearance, although Cyt-PMNs might be slightly more impactful as drivers of associated inflammatory responses.

### Candidiasis

*Candidiasis*, caused by opportunistic human pathogenic *Candida* species, manifests in two forms: mucocutaneous candidiasis and invasive candidiasis, particularly among individuals with suppressed immune status. Among mucocutaneous episodes, oral and vulvovaginal candidiasis are most prevalent, with rough global estimates of 2 million and over >130 million annual cases, respectively^49,50^. Additionally, systemic candidiasis is estimated to affect 700.000 individuals annually worldwide, with mortality rates up to 55%^51^. Other clinically significant but less common clinical forms include chronic hyperplastic candidiasis, noted for persistent lesions with malignant potential, and cutaneous candidiasis, characterized by bright erythema, papulopustules, and satellite lesions^52,53^. These infections can arise from endogenous sources (e.g., own microbiota) or exogenous sources in hospitals, due to improper sterilization methods^54^. The dominant species is *C. albicans*, followed by *C. auris, C. parapsilosis, C. glabrata* and *C. tropicalis*, depending on geographic region and patient population at risk^55^. Notably, candidiasis is the most extensively studied disease with scRNA-seq amongst all fungal pathogens.

#### Systemic candidiasis

##### **Candidemia susceptibility markers and network interplays**

By integrating bulk RNA-seq, genome-wide association studies (GWAS), and scRNA-seq of *C. albicans* (*Ca)*-induced human PBMC samples, another study identified LY86 as a potential candidemia susceptibility marker^56^. The risk allele rs9405943-A specifically drives *LY86* downregulation upon *Candida* stimulation. *LY86* was expressed in both B cells and monocytes, but was selectively downregulated only in monocytes in the presence of *Candida*. Functional validation further demonstrated that reduced *LY86* expression impairs monocyte migration via *CCR2* downregulation, thereby increasing candidemia susceptibility.

A prior meta-analysis of human blood transcriptome studies, integrating scRNA-seq, bulk RNA-seq and microarray datasets, revealed a potential network interplay between TLR and IFN signaling during candidiasis^57^. Using a previous scRNA-seq dataset (obtained from de Vries et al.)^56^, overrepresentation analysis revealed >70 TLR and nearly 100 IFN signaling-associated DEGs across both innate (monocytes, NK cells, pDCs) and adaptive immune cell types (CD4 + , CD8 + , B lymphocytes), with a significant amount (62) overlapping between the two groups. Integrating these scRNA-seq findings with bulk and microarray data, a total of 11 *TLR* and 23 *IFN* genes were confirmed to alter their expression in whole blood cells, PBMCs and moDCs, suggesting that *Candida* infections trigger a consistent response across numerous cell types in blood samples. The meta-analysis also suggests a consistent network interplay between TLR and IFN signaling pathways during systemic candidiasis.

##### **Host-pathogen co-transcriptional dynamics**

The first simultaneous profiling of host and fungal transcriptomic responses using scRNA-seq was performed on *Ca*-infected mouse bone marrow-derived macrophages, at different timepoints^58^. Following infection, time-dependent macrophage and fungal populations were determined. Among macrophages, early responders showed strong proinflammatory signatures (upregulated cytokines, interleukin receptors, transmembrane markers) that markedly reduced in late responders. Differentially expressed Dectin-2 isoforms also distinguished subsets: Dectin-2α (membrane-bound) predominated in early responders, while Dectin-2β (potentially secreted) enriched in late responders. Dectin-2β accumulation might antagonize Dectin-2α signaling and modulate Th17-mediated antifungal responses. Among yeasts, early-stage cells showed enhanced organic acid metabolism, glyoxylate cycle, and transporter activity, while late-stage cells induced carbon/fatty acid metabolism and filamentation, aligning with the yeast-to-filamentous transition during infection. Bimodal expression was also noted for several host (e.g., intracellular pathogen recognition, proinflammation) and fungal genes (e.g., virulence regulatory genes) potentially influencing infection fates.

#### Immune-adrenal crosstalk during systemic candidiasis

##### **Cellular landscapes and functions in the adrenal gland**

During systemic candidiasis, fungal cells disseminate to major organs such as the kidneys, liver, and spleen^59^. Although the adrenal gland is not a primary target of infection, its key role in regulating stress responses—often disrupted in sepsis—prompted one study to examine how its microenvironment alters during infection. Consequently, the study revealed that systemic candidiasis drastically remodels the cellular landscape of murine adrenal tissues with adrenocortical cells^60^. Regarding cellular functions, the accumulating endothelial cells exhibited a proliferation/angiogenesis, MAPK signaling, and migration/adhesion-promoting transcriptional profile, concurrently showing vulnerability to lipid peroxidation. Simultaneously, adrenal macrophages exhibited enhanced effector function, characterized by the upregulation of genes mediating antigen presentation, phagocytosis, ROS production, and inflammasome assembly.

##### **Dysfunctional populations revealed by pseudotime trajectory analysis and cell communication networks**

When further assessing the *Candida*-altered immune-adrenal crosstalk, *Star* (steroidogenic acute regulatory protein) expression levels were used to map ACC transition states^60^. Pseudotime trajectory analysis revealed an active steroidogenic path (high hormone production) and a dysfunctional path (low hormone production). While both trajectories attempted regeneration, they diverged in metabolic and cell-cycle management. Dysfunctional cells exhibited aberrant mitochondrial and cell-cycle activity, suggesting maladaptive proliferation/apoptosis. Consequently, the infection-induced inflammatory milieu might drive ACC loss through the failure/death of the dysfunctional population.

The study also indicated intercellular communication modes. For instance, robust interactions were identified between infiltrating immune and adrenal cells linked by MIF, collagen signaling and complement signaling pathways. Furthermore, NOTCH signaling mediated communication between chromaffin and other cell types, while TGFβ, TNF, and VCAM signaling pathways mediated communication between macrophages and adrenal cells and other immune cell types. Systemic candidiasis thus triggers complex immune-adrenal crosstalk.

#### Cutaneous candidiasis

##### **Skin cellular landscapes and defense mechanisms during anti-*C. auris* responses**

As *C. auris* (*Cau*) is known to effectively colonize the human skin, using scRNA-sequencing, Balakumar et al. aimed to characterize host cell populations responsive to *Cau* in a murine model of cutaneous candidiasis^61^. As a result, various myeloid cell—neutrophil, macrophage, inflammatory monocyte, DC—and lymphoid cell populations—Th, NK, Treg, cytotoxic T, B, proliferating T, γδ T—were shown to accumulate at the infection site. Among non-immune cells, fibroblasts dominated in response to this species with four subtypes.

Subsequent analyses revealed that myeloid and lymphoid cells showed marked inflammatory upregulation, with high cytokine, chemokine, PRR and antimicrobial peptide (AMP) expression. The primary AMP producers were neutrophils and macrophages. T cell subsets displayed pro-inflammatory signatures, including elevated Th17 and stress responses, while mainly NK cells, but also multiple T-cell subsets, mediated antifungal immunity through strong *IFN-γ* expression. Among non-immune cells, fibroblasts were most responsive, enriched in host-defense pathways (including IL-17, HIF-1, PI3K-Akt signaling) and highly expressed AMPs. Other non-immune cells, such as endothelial cells, outer bulge cells, and pericytes, also showed Th17-associated enrichment and produced neutrophil-recruiting chemoattractants. scRNA-seq further revealed immune evasion by *Cau* via macrophage *IL-1Ra* induction, potentially impairing neutrophil function through *IL-1R* blockade.

#### Chronic hyperplastic candidiasis

##### **Identification of disease-determining cell populations and their profiling**

Another study characterized microenvironmental changes in chronic hyperplastic candidiasis (CHC)—a severe form of oral candidiasis—using patient tongue biopsies and revealed a marked enrichment of T/NK cells alongside a depletion of fibroblasts compared to healthy tissues^52^. The study further identified a distinct T cell subpopulation, exhausted CD8 + T cells (CD8+ Tex), and a DC subpopulation, cDC_LAMP3, specifically accumulating in CHC samples. During CHC, fibroblasts exhibited strong functional activation, with increased collagen I production, pro-fibrotic EMT signaling, and matrix remodeling, presumably driving the clinical symptoms of the disease. This was accompanied by upregulated EMT pathways and elevated expression of key extracellular matrix components, including *COL1A1*, *MMP1*, and *MMP2*. These findings obtained through scRNA-seq correlated with CHC’s tongue hardening through collagen deposition and sustained intense inflammation via matrix turnover. Thus, the above fibrotic signatures of fibroblasts might later serve as potential hallmarks of CHC progression.

The accumulating CD8+ Tex cells also displayed elevated exhaustion scores and were characterized by upregulated exhaustion genes and robust PD-1 and TIGIT signaling.

##### **Cell communication networks in CHC**

Despite their reduced abundance in CHC patients, fibroblasts emerged as central orchestrators of intercellular communication^52^, engaging critical ligand-receptor interactions with epithelial, endothelial, and myeloid cells, driving key chemokine signaling pathways essential for immune recruitment, inflammation, and tumor progression. The enriched cDC_LAMP3 subset also interacted extensively with various immune populations. High *PD-L1* expression on these cells facilitated inhibitory crosstalk with CD8+ Tex cells via the PD-1/PD-L1 axis, suggesting they drive T-cell exhaustion and suppress cytotoxic immunity within CHC lesions. Thus, scRNA-seq revealed fibroblasts, cDC_LAMP3 and CD8 + T cells as key mediators of CHC pathogenesis.

### Pneumocystis pneumonia

Pneumocystis pneumonia, caused by *Pneumocystis jirovecii*, is an opportunistic pathogenic infection, occurring in immunosuppressed patients, e.g., HIV + , but also HIV− patients. While early life exposure is common, recent evidence suggests the fungus is cleared; thus, adult pneumonia likely results from recent transmission rather than reactivation^62–64^. Transmitted via airborne particles, the infection manifests as diffuse interstitial pneumonia with an annual worldwide incidence of approx. 500 000 cases and mortality rates up to 30%^49,65^. *Pneumocystis* species exhibit strong host specificity, with *P. jirovecii* being a human-specific pathogen (causing PjP) and unable to infect laboratory animals. Therefore, in vivo models of Pneumocystis pneumonia (PCP) rely on species-specific organisms, such as *P. murina* in mice and *P. carinii* in rats^65–67^. *Pneumocystis* species are difficult to culture axenically^68^, which limits in vitro characterization and leaves many disease features poorly defined. However, recent scRNA-seq studies have proven valuable in elucidating their pathobiology in vivo.

#### Host protective T cell and clonal T cell responses against PCP

By combining scRNA-seq with single-cell TCR sequencing (scTCR-seq), T cell responses were assessed in mice lungs over time, against *P. murina*. The obtained results indicated that among the eight identified distinct T cell subsets, naïve CD4+ and CD8 + T cells gradually decreased while effector CD4 + T cells increased markedly by the fourth week in infected mice^69^. Subsequent sub-clustering of CD4 + T cells revealed transcriptionally distinct subsets, of which naïve CD4 cells showed a marked reduction, while Th1, Th17 and Treg populations significantly increased after the infection. Clonal expansion predominated in the CD4+ compartment—specifically within Th17 and mixed effector clusters—but was also observed in CD8+ and dividing cells. The clonally expanded CD4+ cells showed enhanced cell adhesion, cytokine signaling and upregulated inhibitory receptors (Pdcd1, Lag3, Ctla4). Th17 cells further exhibited a tissue-resident memory (TRM)-like Th17 phenotype previously linked to defense against bacterial pneumonia^70^. The clonally expanded CD8 + T cell population displayed a potent cytotoxic signature marked by *NKG7* and granzymes. Thus, the study highlighted the emergence of activated Th and cytotoxic T cell responses in lungs during PCP.

#### Mapping of the Pneumocystis life cycle in vivo

Through scRNA-seq and pseudotime trajectory analysis, a previous study bypassed cultivation barriers to map *P. carinii* life cycle. scRNA-seq profiling of *P. carinii* from rat BALF identified 13 transcriptional clusters (C1–C13) spanning trophic, mating, and sexual stages, with pseudotime analysis outlining progression from early to mature asci^71^. Early clusters were metabolically active trophic forms; intermediate clusters reflected mating competence (with C7 as a transition point); late clusters captured meiotic initiation to ascus maturation. Three ascus-specific genes (*T552_01968 (mcp1), T552_01043 (dmc1), T552_01932*) were further enriched in the late-stage form (mature asci) and absent in earlier stage forms or after anidulafungin treatment. Because sexual-phase asci drive host-to-host transmission, these genes also represent potential biomarkers for PCP. Therefore, the high-resolution transcriptomic map obtained in this study confirmed the occurrence of sexual reproduction in this host-obligate pathogen.

## Plant—pathogenic fungal infections

Plant fungal diseases represent a significant risk to global food security and long-term agricultural sustainability, indirectly affecting public health and livelihood. As the greatest biotic challenge to global agriculture, fungal diseases destroy up to 23% of crops pre-harvest, and another 20% post-harvest. This annual loss represents enough food to feed 4 billion people, with a 2000-calorie diet, for a year^3^, constituting a serious global humanitarian issue. These diseases include stalk rot (affecting maize, sorghum), head blight (wheat, barley), and rice blast (rice, other small grains). To improve the development of antifungal treatments, scRNA-seq studies of fungal phytopathologies have mapped tissue-specific diversity and uncovered key disease mechanisms. The interaction between *Arabidopsis thaliana*—a common model of plant biology—and *Colletotrichum higginsianum* a hemibiothrophic fungus, causing anthracnose—was also examined, to provide an overview of plant immunity.

### Anthracnose

*Colletotrichum* spp. are hemibiotrophic fungi that cause anthracnose in cruciferous crops, characterized by small, dark, sunken lesions on leaves, stems, or fruits^72^. In warm, humid tropical and subtropical regions, e.g., in Sub-Saharan Africa, Central and South America or South Asia, unmanaged anthracnose is economically devastating, potentially causing crop losses of 30% to 100%, although the disease occurs on nearly every continent^73^. Amongst the species within this genus, *Colletotrichum higginsianum* (*Ch*) is the most frequently used species to model disease development and progression.

#### NLR receptors and GSL metabolism in cell-type-specific defense mechanisms

To examine plant immunity to *Ch*, *Arabidopsis thaliana* leaf protoplasts were analyzed with scRNA-seq after infection^74^. Inoculated and mock-treated leaf comparisons revealed shifts in the cellular landscape specifically during late-stage infection. Among the identified clusters, mock samples predominantly contributed to mesophyll clusters, whereas the late infection-time-point samples were enriched for mesophyll clusters, an epidermal cluster, and two clusters of unknown identity (later assigned to 4 cell types). Furthermore, assessment of intracellular immune receptor classes of NLRs unveiled their enrichment in the vasculatures. Specifically, TNLs were predominantly expressed in procambium cells, RNLs displayed cell-type-specific patterns, and CNLs showed no consistent trend in expression. Additionally, GSL metabolism—critical for pathogen resistance—was also shown to be upregulated at the infection site, with IG-specific genes in epidermis/vasculature/guard cells, and core GSL biosynthesis genes in guard/vasculature cells.

#### Potential markers and spatiotemporal dynamics of the infection

In search of GSL metabolism regulatory genes, scRNA-seq identified two regulators excessively involved in anti-*Ch* host responses: *MYB51*, expressed in vasculature and *MYB122* in epidermal cells. Using loss-of-function mutant strains, *MYB122* was validated as a potential disease progression biomarker, due to the severe infection spread and symptoms observed in mutant cells during infection^74^. Host response dynamics revealed that cells nearest to the fungus showed the strongest *FRK1* expression and high activity in antimicrobial secretory pathways. Nearby guard cells upregulated sulfur, nitrogen, and propionate metabolism, while coordinated ABA signaling changes likely induced stomatal closure.

These results indicate substantial, cell-specific transcriptomic reprogramming in *A. thaliana* during late-stage *Ch* infection.

### Stalk rot

Stalk rot, responsible for maize stem collapse, is primarily caused by *Fusarium verticillioides* (*Fv*), with global yield loss ranging from 10 to 50% or up to 100%, depending on region^75,76^. Besides the significant crop loss threatening global food security, the accumulated mycotoxins produced by *Fusarium* sp. in grains pose significant health issues in animals and humans, due to their immunotoxic, neurotoxic or cytotoxic effects^77^. As a primarily soil-, but also seed-, and air-borne pathogen, *Fv* spreads systemically from roots or seeds, as well as through silks and kernel cracks^78^.

#### Transcriptional landscapes and immune-regulatory networks

When examining root defense in resistant (Qi319) and susceptible (B104) maize lines against *Fv*, seven major pathogen-responsive root cell types were identified, with 6 conserved *Fv*-responsive DEGs upregulated in B104 and downregulated in Qi3019^79^. In general, Qi319 exhibited a more robust response. Furthermore, in Qi319, most of the 32 known plant-disease genes exhibited altered expression. Of these, genes of phenylpropanoid biosynthesis, ROS scavenging, and jasmonate defense were all downregulated across most cell types. Further enrichment analysis identified lignin and flavonoid biosynthesis as primary drivers of antifungal responses in all root cell types. By integrating cell-type specific transcriptomes with resistant genes, validated genes and QTL/QTN loci data, the study also revealed six specialized immune-regulatory networks influencing anti-*Fv* responses: root cap/epidermis phenylpropanoid, cortex flavonoid, cortex/metaxylem ETI, stele, RAM/metaxylem IAA–SA crosstalk and endodermis/root cap ICS–SA in a genotype-specific manner.

This high-resolution map of maize root immune networks provides a roadmap for future research on crop immunity.

### Fusarium head blight

Fusarium head blight (FHB), primarily caused by *F. graminearum* (*Fgr*), ranks among the most economically devastating diseases of wheat and barley due to contamination by mycotoxins, poor crop quality or significant yield loss between 10–30% worldwide or up to 100% in susceptible regional cultivars^80–82^. The fungus spreads via conidia or ascospores, germinating on florets or seedlings under warm, humid conditions. Hyphae penetrate through floret surfaces or natural openings/wounds. Once inside, *Fgr* colonizes tissues, causing symptoms like bleached spikelets and premature senescence^83^.

#### *Fgr* delays wheat defenses

Using scRNA-seq, a previous study revealed pathogen adaptation strategies of *F. graminearum* (*Fgr*) responsible for Fusarium head blight^84^. By using the non-host specific pathogen *F. oxysporum f. sp. cubense* (*Foc*), the study identified host responses potentially suppressed by the host-specific pathogen in wheat coleoptiles. Integrating multiple datasets, cell clusters were mapped to coleoptile layers, and among the eight major cell types identified, phloem and outer sheath cells responded rapidly, but only against *Foc*, not *Fgr*. *Fgr* effectively delayed primary defenses, such as the expression of PRRs, ROS burst enzymes and Ca-signaling genes in the phloem, while photosynthesis, water transport, responses to heat and hydrogen peroxide and amino-acid-related metabolic processes in the outer sheath. In addition, in the outer sheath, antimicrobial phytoalexin biosynthetic genes (HCAA) also remained silent during *Fgr* infection. *Fgr* also induced a less compartmentalized immune activation compared to *Foc*. Outer sheath lignification resisted *Foc* but failed against *Fgr* due to delayed dirigent expression. Only *Fgr* induced strong parenchyma and epidermis responses. Thus, excessive parenchyma lignification correlated with *Fgr* susceptibility.

#### Pseudotime trajectory analysis reveals *Fgr*-driven mesophyll changes

*Fgr* also reshaped the mesophyll by decreasing parenchyma and expanding outer-sheath cells. RNA velocity and pseudotime trajectory analyses identified three trajectories—two shared between *Fgr* and *Foc,* and one *Fgr*-specific^84^. Across the trajectories, parenchyma cells gradually adopted outer-sheath-like immune traits. Core defense genes upregulated across the branches, with pathogen-specific activation of the *TaACT* (*Foc*) and *TaFROG* (*Fgr*) resistance genes. Additionally, chlorenchyma cells responded differently to *Fgr* and *Foc* early in infection, showing opposing photosynthetic activities. Further analyses revealed a bifurcated trajectory, where *Foc*-infected cells predominated in the photosynthetically-active branch, while *Fgr*-infected cells were enriched in the photosynthetically-depressed branch.

Overall, scRNA-seq showed that *Fgr* evades wheat immunity by delaying defense activation and disrupting the compartmentalized response.

### Rice blast

Rice blast, caused by *Magnaporthe oryzae* (*Mo*), affects rice crops globally with yield losses of 10-30% annually or up to 100% in localized susceptible areas, and threatens food security for approx. 60 million people^85^. *Mo* infects all above-ground plant parts or rice through a specialized appressorium structure that generates enormous turgor pressure to penetrate the plant cuticle, deploying an extensive arsenal of effector proteins to establish infection^86^.

#### Defenders and defense mechanisms against *M. oryzae*

A combination of spatial transcriptomics with scRNA-seq (specifically, single-nucleotide RNA-seq), on *Mo*-infected rice leaves, revealed that procambium cells were most enriched at the site of infection and at the late infection stages^87^. However, PRR and NLR levels suggest mestome sheath cells as primary responders in rice immunity. Besides procambium cells and mestome sheath cells, xylem, large parenchyma and fiber cells also showed increased PRR levels, suggesting broad-spectrum immune activation over time. scRNA-seq also revealed uniform SA (salicylic acid) and JA (jasmonic acid) signaling— involved in disease resistance—across cell types, primarily during late-stage infection. This hormonal shift—where SA dominates at 24 hpi before JA signaling rises at 48 hpi—likely reflects the pathogen’s biotrophic-to-necrotrophic transition. Additionally, late-stage procambium cells activated diterpenoid metabolism, specifically Momilactone A biosynthesis, contributing to rice blast resistance.

#### Pseudotime trajectory analysis defined procambium and epidermal responses

Pseudotime trajectory analysis of procambium cells suggested early enrichment of photosynthesis genes followed by oxidative stress and oxoacid metabolism genes as the disease progressed, and the activation of potassium transport and isoprenoid biosynthesis (momilactone A phytoalexin) at the late infection stage—all to constrain *Mo* invasion^87^. Interestingly, *Mo* suppresses host protective genes at infection sites, notably the potassium transporter *OsHKT9*, which declines locally (but remains induced distally), supporting local fungal proliferation. Pseudotime trajectory analyses also suggest progressive epidermal immune activation—from early Pattern Triggered Immunity (PTI) and phosphorylation signaling to metabolic and oxidative stress—prompting further analysis of MAPK dynamics.

In summary, snRNA-seq indicates that *Mo*-infected rice leaves initiate transient, cell-specific PTI.

### The “Gray Area”

**Fig. 2:**
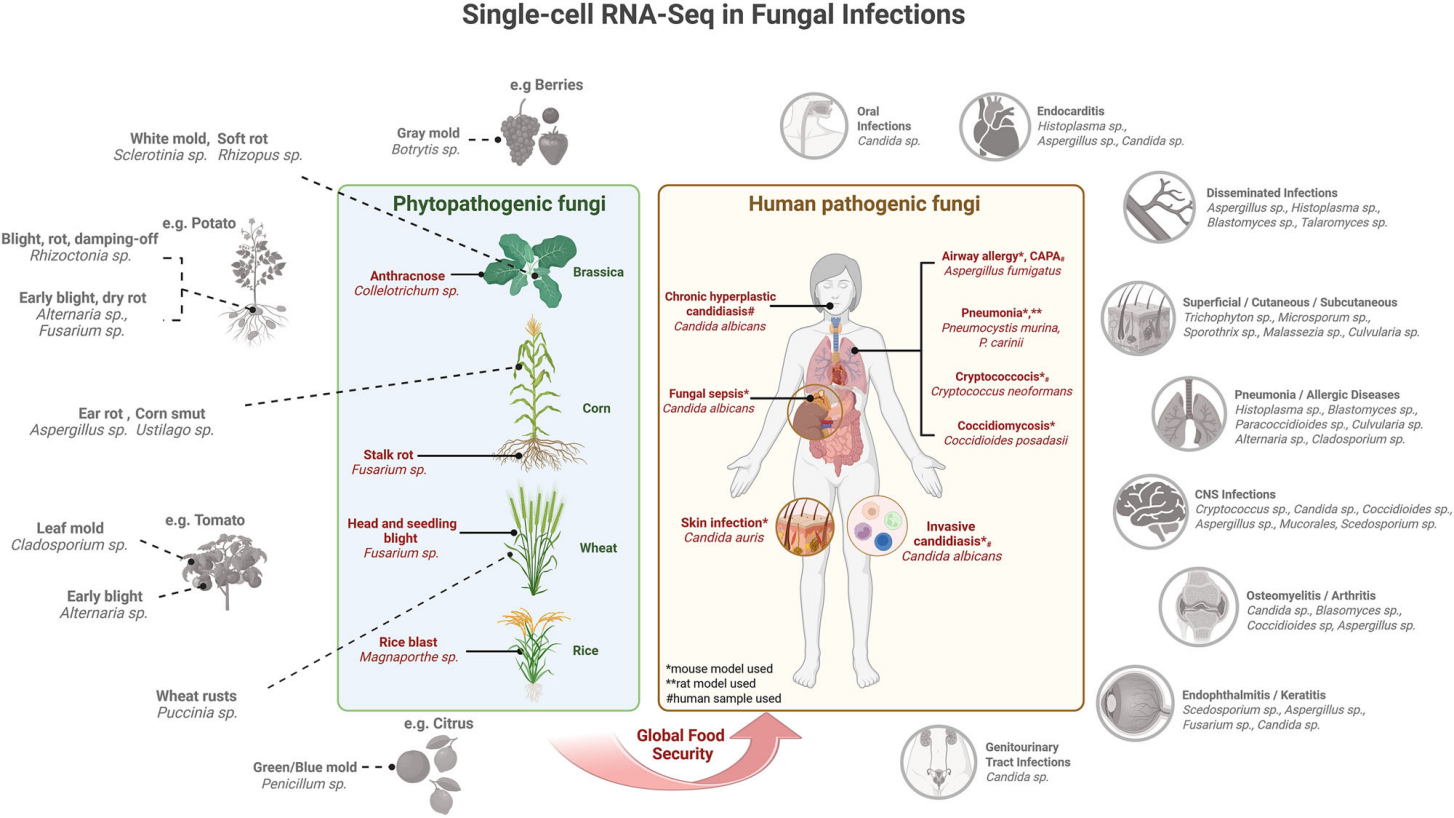
Single-cell RNA-Sequencing in fungal infections. The figure summarizes scRNA-seq studies applied to unveil host-fungal pathogen dynamics during plant and human infections at the single-cell level. Gray areas with dashed lines indicate various fungal genera frequently associated with specific diseases that remain unexamined in the context of host–pathogen interactions. Figure 2 was created in BioRender. Gacser, A. (2026) https://BioRender.com/zji0bon.

While scRNA-seq has advanced our understanding of fungal disease progression, current research represents only the “tip of the iceberg”. As shown in Fig. 2, numerous human and phytopathogenic genera still remain in the “gray area,” lacking single-cell profiling.

For instance, several of the clinically relevant human pathogenic fungi associated with endocarditis, pneumonia, CNS inflammation, and cutaneous infections still warrant attention. For example, *Histoplasma capsulatum*, which causes histoplasmosis, primarily affects immunocompromised individuals in Africa and the Americas^88^ or *Scedosporium sp*., causing fatal abscesses and ventriculitis following near-drowning incidents^89^. *Mucorales sp*. target immunocompromised patients with mortality rates up to 80% despite antifungal and surgical interventions, and their mycotoxin, mucoricin, facilitates angioinvasion^90,91^. *Blastomyces dermatitidis* induces pulmonary infections in North America, potentially disseminating to skin and bones^92,93^, while *Talaromyces marneffei*, endemic in Southeast Asia, primarily affects immunosuppressed individuals through occupational crop/livestock exposure in rainy seasons^94–97^. *Paracoccidioides sp*. cause pulmonary and mucocutaneous infections, especially in rural workers in Central and South America^98^, whereas *Trichophyton* and *Microsporum sp*. induce dermatophytoses, or ringworm^99–102^. *Curvularia* and *Fusarium sp*. can cause localized infections (eye, skin) in tropical, subtropical or temperate regions, but may also become invasive^103–105^. Finally, *Alternaria* and *Cladosporium sp*. are major respiratory threats, exacerbating asthma and allergic diseases worldwide^106–108^.

Similarly, numerous agriculturally relevant phytopathogenic fungi also need to be investigated to enhance crop defense. While *Botrytis cinerea* (gray mold) causes significant pre- and post-harvest fruit losses^109,110^, *Sclerotinia sclerotiorum* (white mold) affects dicots like rapeseed and soybean, producing toxic sclerotia^111,112^. *Rhizopus stolonifera* induces post-harvest soft rots^113^, and *Rhizoctonia solani* damages legumes, cereals, and tubers^114,115^. *Ustilago maydis* leads to corn smut and human infections^116,117^, whilst *Cladosporium fulvum* (tomato leaf mold) infects tomato leaves, causing chlorotic lesions and reduced yield^118,119^. *Penicillium sp*. causes citrus and pome molds, and produce toxins (patulin)^120,121^, while *Puccinia triticina* induces wheat leaf rust epidemics^122^. Finally, *Alternaria solani* and *Alternaria alternata* induce early blight in potatoes and tomatoes^123,124^.

Taken together, investigating these ‘gray areas’ is crucial for uncovering the cellular heterogeneity and markers needed for targeted interventions.

### Gaps and Limitations

Understanding the genetics of pathogenic fungal infections is crucial for elucidating gene function, identifying virulence determinants, and discovering therapeutic targets. However, most scRNA-seq studies of host–fungal interactions focus on the host rather than the pathogen, primarily with the help of 10x Genomics. Fungi in general are underrepresented in transcriptomic datasets −2.8% of bulk and 0.3% of scRNA-seq studies—with research skewed toward *Saccharomyces cerevisiae*, leaving pathogenic fungi scarcely investigated^6^. The host-centric focus is understandable, as fungal pathogens evolve rapidly during infection, while host genotypes remain relatively stable, making defense-centered analyses more feasible.

Fungal genetic research faces significant biological and technical barriers in general, due to thick cell walls, dimorphism, complex life cycles, and ploidy shifts^5^. In scRNA-seq, the fungal cell size also limits transcript capture, increasing data dropout and sparsity, which complicates downstream analysis^5^.

Focusing solely on scRNA-seq, limitations occur mainly during the initial steps. Optimized dissociation—often combining mechanical and enzymatic cell wall disruption—or gradient centrifugation is needed to maximize yield and viability, but may also introduce transcriptomic stress artifacts. The chosen single-cell isolation method (FACS, MACS, LCM) also impacts cell purity and recovery. Additionally, limited or incomplete fungal reference genomes further constrain analysis^6^. Nevertheless, recent advances in scRNA-seq methods—including mDROP-seq, yeastDrop-seq, and SCRB-seq—offer scalable, cost-effective workflows for fungal transcriptomic studies incorporating optimized lysis protocols, higher sensitivity and reduced stress risk, while sharing droplet-based principles of 10x systems^5,6,19,125–127^. While developed for yeasts, they provide a methodological foundation also for filamentous fungi, although hyphal clogging of microfluidics remains a challenge.

Additional limitations reflect general scRNA-seq challenges: the need to study certain pathogens within host-dependent or difficult-to-culture systems can constrain experimental design and biological interpretation, enzymatic dissociation may distort cell composition or remove fragile cells, and precise sampling is critical in dynamic host–pathogen interactions, though often limited by cost and cohort size.

Besides considering these limitations, future studies should also include multiple pathogenic strains, age- and sex-balanced controls, and more functional or human-based validations to enhance translational relevance.

And importantly, while scRNA-seq generates overwhelming amounts of data, more data does not automatically translate into a deeper understanding. Focusing on answering clear biological questions with proper contextualization helps ensure that large descriptive studies are replaced by concise and interpretable insights.

### Future perspectives

While this review focused on scRNA-seq in host–fungal interactions, several aforementioned studies have also integrated complementary sequencing technologies to enhance their datasets (Table 1). Methods such as bulk RNA-seq^56,57,74,79^, scTCR-seq^70^, spatial transcriptomics^87^, and GWAS^56^ have also been used either to provide comparative benchmarks or to offer multi-dimensional perspectives. Furthermore, it is anticipated that other emerging single-cell-based technologies will soon become indispensable tools in further refining our understanding of this field.

One significant category includes single-cell epigenomic methods applicable across cell types and host species. For example, scATAC-seq (single-cell Assay for Transposase-Accessible Chromatin with sequencing) profiles chromatin accessibility, while single-cell bisulfite sequencing maps DNA methylation^128,129^. Additionally, single-cell techniques using specific antibodies linked to protein A-micrococcal nuclease (scChIC) or protein A-Tn5 transposase (scCUT&TAG) assess single-cell histone modifications, and the newly developed single-cell Epi2-seq simultaneously detects DNA methylation and histone marks in the same cell^130,131^. Though not yet widely adopted in immunology, these approaches may reveal the epigenetic bases of cellular heterogeneity in host-fungal interactions.

Another category includes receptor sequencing methods. Similarly to the aforementioned scTCR-seq, scBCR-seq enables analysis of B-cell receptor diversity, specificity and cellular responses at the individual cell level, thereby offering valuable insights into the role of the adaptive immune system in antifungal responses^132^. Overall, integrating these complementary single-cell and sequencing approaches promises a more comprehensive understanding of host–fungal interactions, bridging transcriptomic, epigenomic, and immune receptor perspectives to uncover mechanisms of cellular heterogeneity, immune specificity, and regulatory control.

## Conclusion

This review aimed to highlight the growing application of scRNA-seq in fungal infection research, an area that has historically received less attention in infectious disease research. Here, we summarized key findings and insights, while also identifying knowledge gaps and suggesting future directions for research on host-fungus dynamics. Building on the above studies as set examples summarized in Table 1, we hope this review will encourage the broader adoption of single-cell technologies to enhance research in the field of fungal infectious diseases.

### Reporting summary

Further information on research design is available in the Nature Portfolio Reporting Summary linked to this article.

## Supplementary information

Reporting summary
